# A Key to the Theory of Opsonins

**Published:** 1906-09-22

**Authors:** 


### A KEY TO THE THEORY OF OPSONINS.

THIS subject is so obscured by technical phrases that it is at first somewhat confusing to those unfamiliar with modern research. We therefore propose giving a brief and simplified outline of the general principles of Immunity as held by Metchnikoff and the Opsonin theory of Wright and Douglas.

Metchnikoff holds that the leucocyte is the only element in the blood concerned in the phagocytosis of micro-organisms, and that the blood fluid takes no active share in the process.

The scheme of Metchnikoff's views may be graphically represented in tabular form.

| | | | | |
|---|---|---|---|---|
| Phagocytes | Circulating | Leucocytes | Microphages producing Microcytases | Bacteria + |
| | | | | Cells − |
| | | Lymphocytes | Macrophages producing Macrocytases | Bacteria − |
| | Fixed— | Endothelial cells | | Cells + |

From this table it will be seen that immunity is due to the action of phagocytes which are of two kinds, (1) those which circulate in the blood and lymph, and (2) those which are fixed or stationary in the different tissues of the body. These, again, are divided into *microphages* and *macrophages*. The former are represented by the polymorphonuclear leucocytes, which secrete *microcytases* and operating only upon the more swiftly active bacteria such as streptococci and staphylococci, but have little or no influence upon animal cells such as red blood corpuscles. The *macrophages,* on the other hand, such as the large mononuclear lymph corpuscles and the fixed endothelial cells produce *macrocytases,* which specially operate upon animal cells and the more sluggish or slowly active bacteria such as the bacillus of tubercle and leprosy. Metchnikoff also maintains that in acquired immunity the process is somewhat further complicated by the co-operation of microcytases with *fixatives.* Fixatives are substances derived from phagocytes, too, but are not themselves bactericidal, they only render the bacteria more susceptible to the microcytase ; and each variety of micro-organism has its own specific fixative, while microcytases will attack all kinds of bacteria indiscriminately.

Metchnikoff also maintains that the cytases are only free during phagocytosis. Ehrlich, on the other hand, holds the view that cytases are always free in the body fluids.

The Opsonic theory of Wright and Douglas is based upon entirely different premises.

They maintain that there exists in the blood plasma a substance called *opsonin,* which has the power of influencing bacteria in such a way as to render them specially susceptible to phagocytosis, or, to use a familiar simile, to cook them and prepare them ready for eating, so making them more attractive and digestible to the phagocyte, by forming a chemical union with them. They further hold that the plasma has nothing to do with the quality or voracity of the phagocyte.

SCHEME OF OPSONIN THEORY.

| | | | | |
|---|---|---|---|---|
| OPSONINS in Serum. | *Influence.* | BACTERIA. | *So that they are rendered more susceptible to the action of* | PHAGOCYTES. |

Bulloch has further shown that opsonins are highly specific to each variety of micro-organism ; for while the blood of one person may contain sufficient opsonin to combat the staphylococci of an attack of furunculosis, there may be only half enough to neutralise a tubercular infection.

The details of technique which are necessary to demonstrate these phenomena are too complicated for this short explanation ; we will therefore only briefly consider the general principles and outlines upon which a particular opsonin is estimated and employed.

The first essential is to identify and isolate by cultivation the particular bacterium which is responsible for the disease. Let us assume that the case is one of furunculosis in which the staphylococcus pyogenes is the responsible micro-organism.

Mix equal quantities of the *patient's* serum, staphylococcal emulsion, and washed leucocytes in a capillary tube. Seal, and incubate at 37° C. for fifteen minutes. Similarly treat some *normal* serum. Make film, stain, and compare. It will be found that in each tube the phagocytes have absorbed bacteria which can be seen inside them. In that of the patient forty phagocytes will contain about eighty bacteria, the average per leucocyte being $\frac{80}{40}=2$, – the *patient's* PHAGOCYTE INDEX. In the *normal* films we shall find that forty leucocytes will contain as many as 160 staphylococci, giving $\frac{160}{40}=4$ as the *normal* PHAGOCYTE INDEX. This difference is due to the amount of opsonin present in the respective sera ; in that of the patient there is a deficiency of opsonin in the patient's, the ratio being 2 : 4 or 0.5 : 1. The latter figure is the *normal* OPSONIC INDEX. The patient's opsonic index being 0.5 means that he is deficient to the extent of one half the amount of opsonin necessary for complete phagocytosis of the staphylococcal infection.

In the case of tuberculosis the opsonic index is generally 0.7 when the disease is purely localised, but when it is a systematic or diffused infection the index varies considerably, being one day as high as 1.6, at another as low as 0.6.

The opsonin treatment by Professor Wright's method consists of introducing into the patient sufficient standardised vaccine of the dead bacteria from which he is suffering, so as to raise the proportion of antibacterial substance in his circulation and to restore his deficiency.

With regard to tuberculosis, it is further important to recollect that three distinct opsonic phases following an appropriate dose of the vaccine are strikingly exhibited.

(1) The first which follows immediately after inoculation is a well-marked fall or *Negative* phase; this is succeeded by (2) a steady rise of opsonic power from day to day for a week—the flow culminating in (3) the *Positive* phase or "high tide."

Wright accentuates the importance of not inoculating during the negative phase, neither does he advise reinoculation during the second phase of "flow," but waiting for the third or *Positive* phase.

These phases unfortunately cannot be recognised by any of the ordinary chemical phenomena, therefore recourse must be made to an estimation of the opsonic power of the blood, and since this can only be attained by complicated and precise methods, success or even safety in the vaccine treatment of infectious diseases, especially tuberculosis, can at present only be carried out with the assistance of a skilled observer and a properly equipped laboratory.

Note.—For an exhaustive account of the subject an excellent paper by G. W. Ross, Brit. Med. Jour., July, 1906, and Binnie's translation of Metchnikoff's "Immunity to Infectious Diseases," Cambridge, 1905, may be consulted.

